# 147. Antibiotic Prescribing: Shorter is Also Better in the Emergency Department

**DOI:** 10.1093/ofid/ofab466.349

**Published:** 2021-12-04

**Authors:** Lisa Vuong, Darius Faison, Julie Thomson, Rachel Kenney, Susan L Davis, Susan L Davis

**Affiliations:** 1 Henry Ford Hospital, Detroit, MI; 2 Henry Ford Wyandotte, Wyandotte, MI; 3 Wayne State University, Detroit, MI

## Abstract

**Background:**

Published information suggests room for improvement in antibiotics prescribed on discharge from the emergency department (ED). The objective of this study was to evaluate antibiotic prescribing in the ED for uncomplicated infections of the lower respiratory tract (LRTI), urinary tract (UTI), and skin and skin structure (SSTI).

**Methods:**

IRB-approved retrospective cross-sectional study of patients discharged from the ED from January to June 2019 at 6 locations. Inclusion: ≥ 18 years old and uncomplicated LRTI, UTI, or SSTI. Exclusion: hospital admission. Appropriate prescribing was defined having all three of the following correct per local and national guidelines: antibiotic selection, dose, and duration. Correct duration: 5 days for LRTI and SSTI; 3 days for trimethoprim-sulfamethoxazole (TMP-SMX), 5 days for nitrofurantoin (NFT), and 7 days for beta-lactams for UTIs. Endpoints within 7 days: antibiotic escalation, readmission to ED or hospital, any outpatient contact, and report of adverse drug event (ADE). Endpoints within 90 days: *Clostridioides difficile* infection (CDI). Descriptive and bivariable statistics were performed.

**Results:**

Inappropriate prescribing: 77% (304) vs. appropriate 23% (89). Infection type: 47.8% SSTI, 30% UTI, and 22.1% LRTI. SSTI was associated with the greatest proportion of inappropriate prescribing at 89.4% (Figure 1). Comparisons for inappropriate vs. appropriate groups: 15.8% vs. 22.5% for beta-lactam allergy and 23.4% vs. 19.1% for cultures drawn in ED. Most common antibiotics for inappropriate vs. appropriate: first generation cephalosporin at 70.1% vs. 7.3% (p< 0.05), TMP-SMX at 14.3% vs. 12.2% (p=0.75), and NFT at 7.8% vs. 65.9% (p< 0.05). Prescriptions considered inappropriate were primarily driven by excess duration (Figure 2). Endpoints for inappropriate vs. appropriate groups: antibiotic escalation at 6.6% (2.8% were due to cultures drawn in the ED) vs. 1.1% (p=0.06), readmission at 8.6% vs. 9.0% (p=0.9), any outpatient contact at 18.4% vs. 19.1% (p=0.89), and report of ADE at 1.3% vs. 1.1%. No CDI in either group.

Figure 1. Appropriateness of Discharge Prescriptions by Infection Type, N = 393

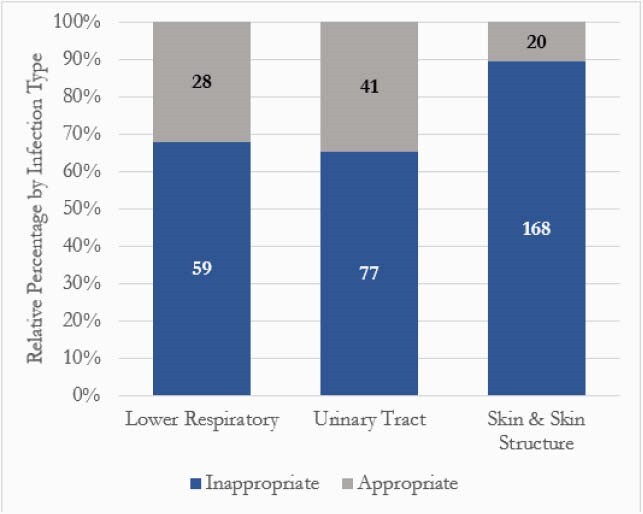

Figure 2. Subset Analysis: Reasons for Inappropriate Prescribing, n = 304

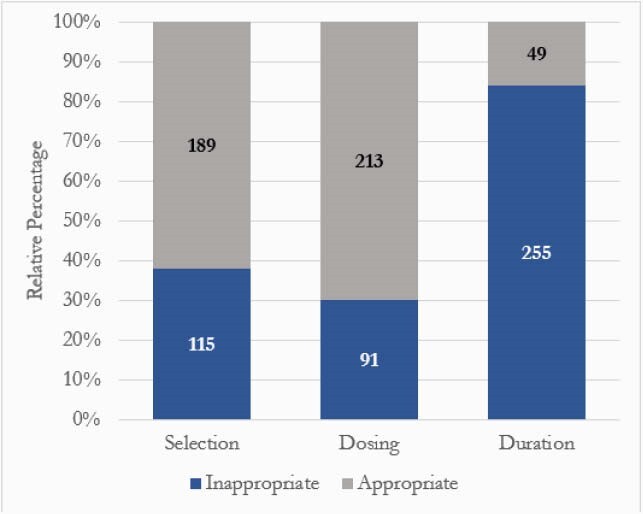

**Conclusion:**

The main reason for inappropriate prescribing in the ED was excess duration of therapy, making this an area of opportunity for future antibiotic stewardship improvement.

**Disclosures:**

**Rachel Kenney, PharmD**, **Medtronic, Inc.** (Other Financial or Material Support, spouse is an employee and shareholder) **Susan L. Davis, PharmD**, Nothing to disclose

